# High-throughput compound screening identifies navitoclax combined with irradiation as a candidate therapy for HPV-negative head and neck squamous cell carcinoma

**DOI:** 10.1038/s41598-021-94259-5

**Published:** 2021-07-20

**Authors:** Katja Tuomainen, Aini Hyytiäinen, Ahmed Al-Samadi, Philipp Ianevski, Aleksandr Ianevski, Swapnil Potdar, Laura Turunen, Jani Saarela, Sergey Kuznetsov, Wafa Wahbi, Maija Risteli, Antti Mäkitie, Outi Monni, Tuula Salo

**Affiliations:** 1 Department of Oral and Maxillofacial Diseases, University of Helsinki, 00014 Helsinki, Finland; 2 Translational Immunology Research Program (TRIMM), University of Helsinki, 00014 Helsinki, Finland; 3 Institute for Molecular Medicine Finland (FIMM), University of Helsinki, 00290 Helsinki, Finland; 4 Cancer and Translational Medicine Research Unit, University of Oulu, 90014 Oulu, Finland; 5 Department of Otorhinolaryngology, Head and Neck Surgery, Helsinki University Hospital (HUS), University of Helsinki, 00029 Helsinki, Finland; 6 Research Program in Systems Oncology, Faculty of Medicine, University of Helsinki, 00014 Helsinki, Finland; 7 Division of Ear, Nose and Throat Diseases, Department of Clinical Sciences, Intervention and Technology, Karolinska Institute, Karolinska Hospital, 141 86 Stockholm, Sweden; 8 Applied Tumor Genomics Research Program, Faculty of Medicine, University of Helsinki, 00014 Helsinki, Finland; 9 Medical Research Center, Oulu University Hospital, 90220 Oulu, Finland; 10 Department of Pathology, Helsinki University Hospital (HUS), 00029 Helsinki, Finland

**Keywords:** Drug discovery, Cancer, Head and neck cancer

## Abstract

Conventional chemotherapeutic agents are nonselective, often resulting in severe side effects and the development of resistance. Therefore, new molecular-targeted therapies are urgently needed to be integrated into existing treatment regimens. Here, we performed a high-throughput compound screen to identify a synergistic interaction between ionizing radiation and 396 anticancer compounds. The assay was run using five human papillomavirus (HPV)-negative head and neck squamous cell carcinoma (HNSCC) cell lines cultured on the human tumor-derived matrix Myogel*.* Our screen identified several compounds with strong synergistic and antagonistic effects, which we further investigated using multiple irradiation doses. Navitoclax, which emerged as the most promising radiosensitizer, exhibited synergy with irradiation regardless of the p53 mutation status in all 13 HNSCC cell lines. We performed a live cell apoptosis assay for two representative HNSCC cell lines to examine the effects of navitoclax and irradiation. As a single agent, navitoclax reduced proliferation and induced apoptosis in a dose-dependent manner, whereas the navitoclax–irradiation combination arrested cell cycle progression and resulted in substantially elevated apoptosis. Overall, we demonstrated that combining navitoclax with irradiation resulted in synergistic in vitro antitumor effects in HNSCC cell lines, possibly indicating the therapeutic potential for HNSCC patients.

## Introduction

Head and neck squamous cell carcinoma (HNSCC) represents the eighth most common malignancy globally^1^. Treatment approaches include surgery combined with radio-, chemo-, immuno- or targeted therapy relying on the epidermal growth factor receptor (EGFR) inhibitor cetuximab (Erbitux). Chemoradiotherapy is a standard treatment for locoregional advanced or unresectable HNSCC. Despite improved treatment options, the 5-year survival rate for HNSCC has remained stagnant, at approximately 50%^2^. Furthermore, current chemotherapeutic agents are nonselective and accompanied by severe side effects. Combinations of cetuximab or chemotherapy with irradiation appear to improve patient survival, although response rates to cetuximab remain low^3^. Therefore, new molecular-targeted therapies, which can be integrated into existing treatment regimens, are urgently needed.

Current in vitro anticancer compound testing carries a low predictive value, since only 5% of compounds demonstrating efficacy in preclinical tests have been approved following clinical trials^4^. These tests are often conducted on a two-dimensional (2D) plastic surface or using animal-derived extracellular matrices, such as Matrigel, thereby overlooking the important interaction between cancer cells and the human tumor microenvironment (TME). To overcome this problem, we have developed a human tumor leiomyoma-derived matrix, Myogel, to more effectively mimic the human TME^5, 6^. Previously, we demonstrated that using Myogel-coated wells improved the predictability of in vitro anticancer compound testing compared to conventional plastic and animal-derived matrix-coated wells^7^.

Ionizing radiation induces double-strand DNA breaks, while unsuccessful repair halts the cell cycle or leads to apoptosis. One cancer hallmark is the ability of tumor cells to resist cell death^8^, both critical in carcinogenesis and representing a major obstacle to effective treatment^9^. One suggested mechanism of resistance to anticancer treatment lies in the altered expression of B-cell lymphoma 2 (Bcl-2) family members^9^. The Bcl-2 family proteins, such as Bcl-2 and Bcl-xL, control cell death by regulating the mitochondrial outer membrane permeability, allowing for the release of intermembrane proteins to the cytoplasm and caspase activation leading to apoptosis^10, 11^. Inactivating the mutation of tumor suppressor p53 is the most frequent genomic alteration in HNSCC^12^. Functional p53 directly and indirectly inhibits Bcl-2^13^, rendering Bcl-2 a potential therapeutic target in HNSCC. Navitoclax (ABT-263), a potent and selective inhibitor of Bcl-2/Bcl-xL, has demonstrated in vitro and in vivo activity against a large panel of cancer cell lines, such as small-cell lung cancer and hematologic malignancies^14, 15^. However, only a few in vitro studies exist for HNSCC^16, 17^. To our knowledge, in vitro studies regarding the presence of the extracellular matrix combining multiple irradiation doses and navitoclax remain unreported.

This study aims to identify new anticancer compounds with radiosensitizing properties for HNSCC. We performed high-throughput compound screening (HTS) on five HPV-negative HNSCC cell lines cultured in the human tumor–derived matrix Myogel. Interestingly, several anticancer compounds demonstrated strong synergistic or antagonistic effects when combined with ionizing radiation. To further investigate the synergistic interaction among the best-performing hits, we combined the anticancer compounds and ionizing radiation in a pairwise dose–response manner and tested the combinations in 6 × 5 dose–response matrices. Navitoclax, as the most promising radiosensitizer, was further examined using an additional eight HPV-negative HNSCC cell lines. The live cell apoptosis assay on two HNSCC cell lines confirmed that navitoclax substantially increased irradiation-induced apoptosis.

## Results

### HTS of 396 anticancer compounds reveals synergistic and antagonistic combinations with ionizing radiation on HNSCC cells

High-throughput compound screening (HTS) is a widely used method for identifying effective drug candidates targeting cancer cells. We used a compound library of 396 FDA-approved drugs as well as experimental drug candidates and probes together with ionizing radiation to investigate potential synergistic and antagonistic combinations in five locally established HNSCC cell lines with previously characterized mutation profiles^18^.

**Figure 1:**
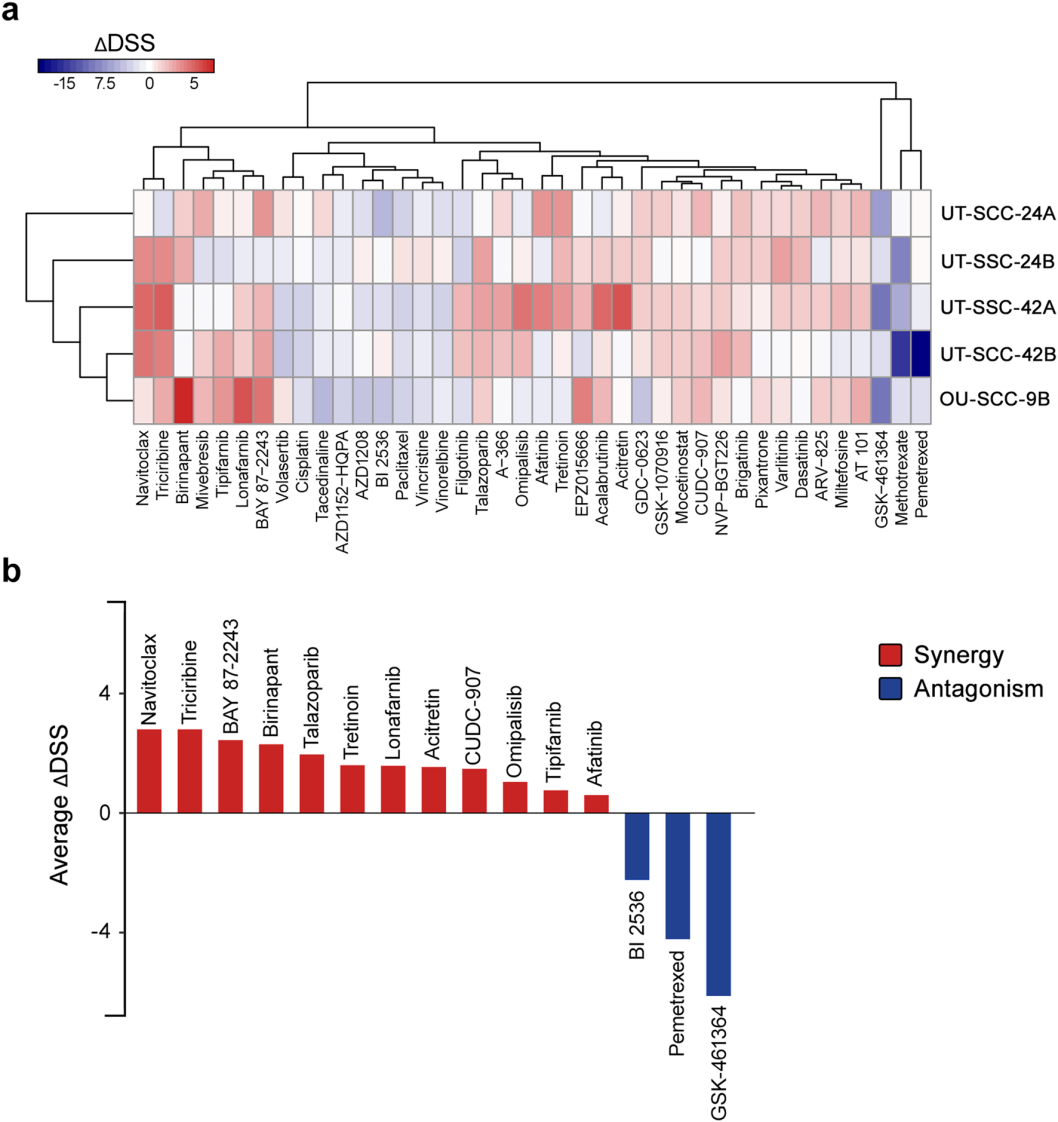
A high-throughput drug screen with an EC20 irradiation dose reveals the potential synergistic and antagonistic properties of the drugs. **(a)** A library of 396 experimental and FDA-approved drugs was tested against five HNSCC cell lines with or without irradiation (top 40 compounds with the highest absolute median values are shown), depicting the relative cell viability quantified as selective drug sensitivity scores (ΔDSS). Red and blue areas, respectively, indicate the potential synergistic and antagonistic combinations with irradiation. The full heatmap appears in Supplementary Figure S1. **(b)** Average ΔDSS of compounds exhibiting the most promising synergistic and antagonistic properties.

The compound screening revealed a remarkable variation across the five HPV-negative HNSCC cell lines in their response to most compounds and compound–irradiation combinations (Fig. 1a and Supplementary Figure S1). However, some compounds were completely inactive (n = 39) both as a single agent and combined with irradiation across all five cell lines (Supplementary Figure S1). The other group of compounds appeared highly effective as a single agent in all five cell lines, but exhibited an additive effect when combined with irradiation (n = 28). The majority of the compounds exhibited a synergistic or antagonistic interaction when combined with irradiation (n = 357), although most lacked consistency across cell lines. Nevertheless, some compounds exhibited a clear synergistic or antagonistic pattern across all cell lines. Based on the average ΔDSS values, we selected the 15 most promising synergistic (n = 12) and antagonistic (n = 3) compounds for further validation (Fig. 1b). Interestingly, 7 of 12 synergistic compounds were classified as differentiating or epigenetic modifiers (BAY 87-2243, talazoparib, tretinoin, lonafarnib, acitretin, CUDC-907 and tipifarnib). Three synergistic compounds were kinase inhibitors (triciribine, omipalisib and afatinib) and two were apoptotic modulators (navitoclax and birinapant). Three antagonistic compounds were PLK1 kinase inhibitors (BI 2536 and GSK-461364) and the metabolic modifier pemetrexed.

### Top-hit validation in dose–response matrices revealed strong antagonism and synergy in several drug–irradiation combinations

To validate the most promising combinations, we performed a comprehensive drug combination screen of 15 compounds and multiple irradiation doses measured in 6 × 5 dose–response matrices using the same HTS protocol used in the initial screen. To test whether the combinations acted synergistically or antagonistically, we compared the observed responses to the expected combination responses based on the ZIP reference model using the SynergyFinder web application (version 2)^20, 21^. Based on the obtained synergy scores, we classified the compound–irradiation combinations as additive, antagonistic or synergistic. Combinations with a synergy score > 10 were considered strongly synergistic and those < − 10 were considered strongly antagonistic, while synergy scores between − 5 and 5 were classified as additive. Other synergy scores represented borderline synergistic/antagonistic drug pairs.

**Figure 2:**
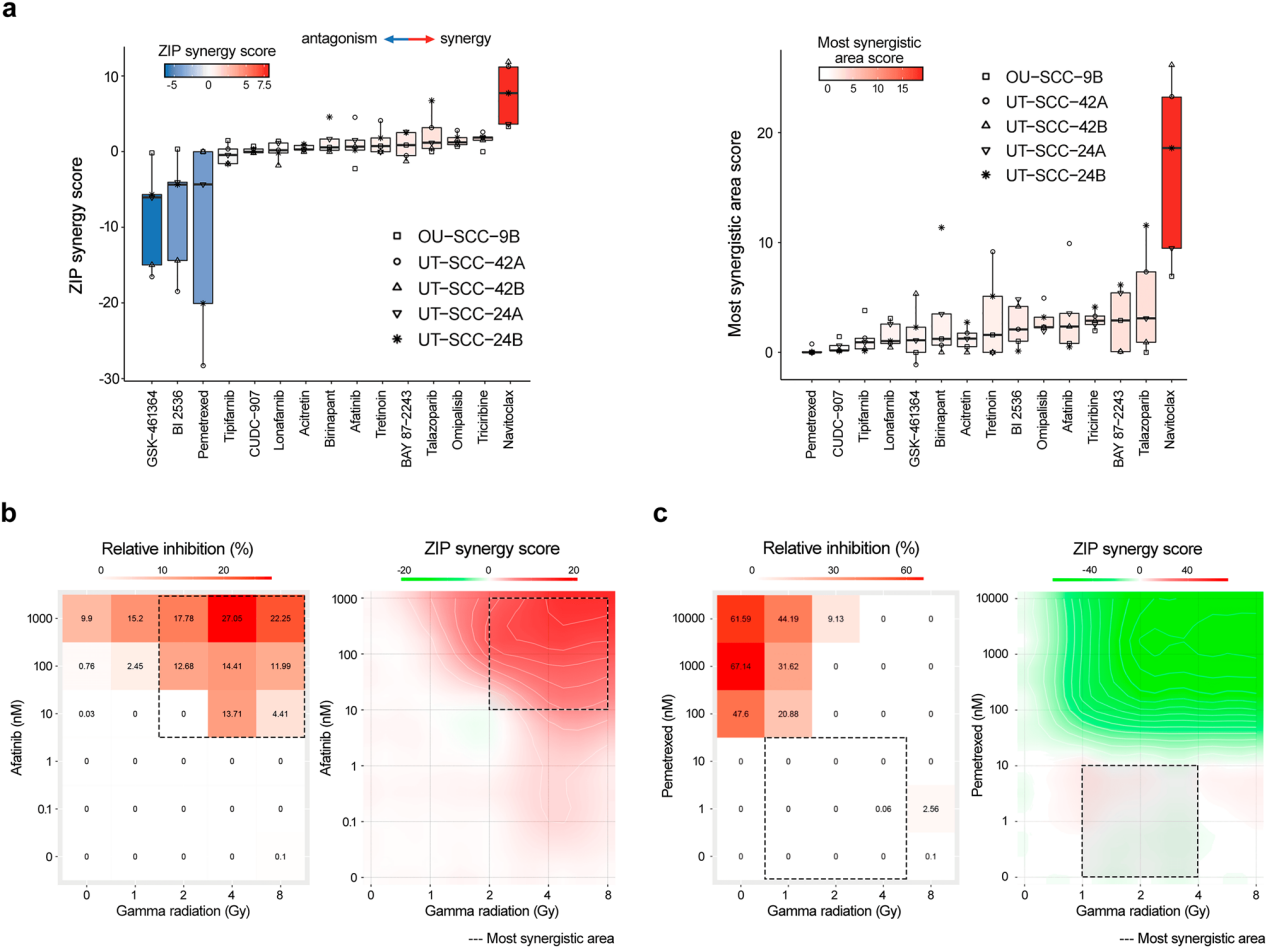
The synergy validation for the 15 most promising combinations tested in HNSCC cell lines grown on Myogel. **(a)** Quantification of combination synergy across the five HNSCC cell lines using two commonly used synergy metrics: average synergy score (left panel) and most synergistic area score (right panel) in the SynergyFinder software. **(b)** Representative example of synergistic afatinib–irradiation combination tested on the UT-SCC-42A cell line. The dose–response matrix showing CellTiter-Glo (CTG) viability at different dose pairs (left) and synergy distribution plot calculated based on the ZIP synergy reference model (right) are shown. **(c)** Representative example of antagonistic pemetrexed–irradiation combination tested on the UT-SCC-42A cell line. The dose–response matrix showing CTG viability for different dose pairs (left) and the synergy distribution plot calculated based on the ZIP synergy reference model (right) are shown. The most synergistic area score represents a synergy score calculated for the most synergistic 3 × 3 dose window (dashed rectangle).

The dose–response analysis verified that, among seven tested epigenetic modifiers, only two (talazoparib and tretinoin) exhibited a promising synergy (Fig. 2a). Only one of three kinase inhibitors, afatinib (EGFR inhibitor), was also validated to exhibit synergy (Fig. 2b). Both apoptotic modulators (navitoclax and birinapant) also exhibited a synergy when combined with irradiation similar to the previous screen. Interestingly, the SMAC mimetic antagonist birinapant showed synergy only in one cell line (UT-SCC-24B), whereas navitoclax (Blc-2 and Bcl-xl inhibitor) exhibited synergy across all five cell lines (Fig. 2a and Supplementary Table S1). In addition, we validated that PLK1 kinase inhibitors (BI 2536 and GSK-461364) and the metabolic modifier pemetrexed exhibited a strong antagonism when combined with irradiation, similar to results from the initial screen (Fig. 2a,c).

### Navitoclax and irradiation exhibited synergy regardless of p53 mutation status

Navitoclax exhibited the highest synergy when combined with ionizing radiation among the tested compounds (Fig. 2a). A strong synergy (> 10) was observed in three of five cell lines (UT-SCC-42A, UT-SCC-42B and UT-SCC-24B) and moderate synergy (5–10) in two other cell lines (UT-SCC-24A and UT-SCC-9B; see Fig. 2a, right panel).

**Figure 3:**
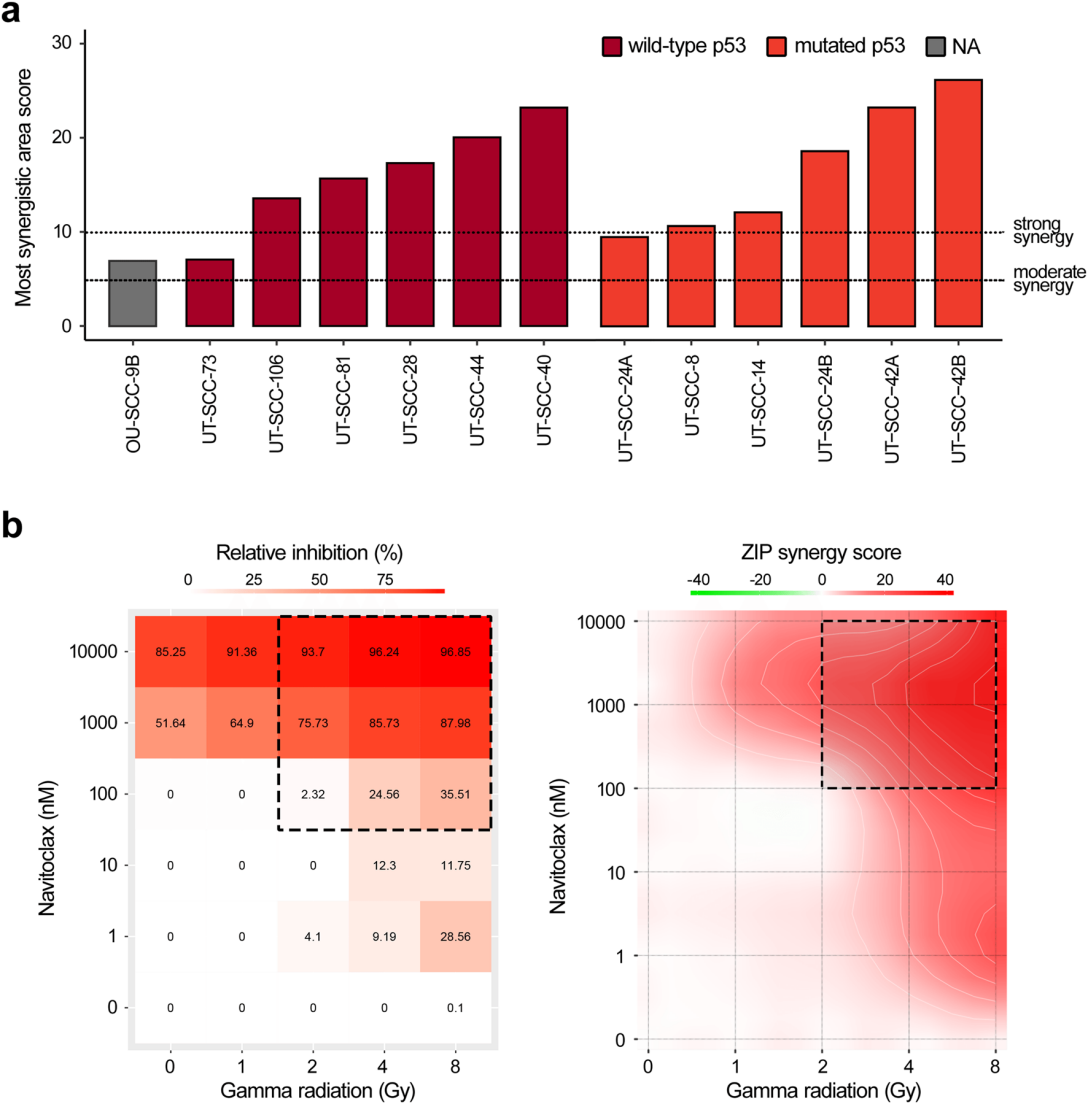
The combination of navitoclax and irradiation exhibited a strong synergy regardless of the p53 mutation status. **(a)** The most synergistic area scores for the navitoclax–irradiation combination across 13 HNSCC cell lines. **(b)** A representative example of the navitoclax–irradiation combination tested on the UT-SCC-40 cell line. The dose–response matrix showing CTG viability for different dose pairs (left) and the synergy distribution plot calculated based on the ZIP synergy reference model (right) are shown. The most synergistic area score represents a synergy score calculated for the most synergistic 3 × 3 dose window (dashed rectangle).

Next, we performed an extended navitoclax–irradiation dose–response screen on an additional eight HNSCC cell lines (Fig. 3a). In total, we tested 13 cell lines, consisting of six p53-mutated, six wild-type and one uncategorized cell line. The HNSCC cell line mutation profiles were characterized in a previous study^18^. We observed a strong combination synergy across ten cell lines and a moderate synergistic effect in three other cell lines (Fig. 3a). Therefore, we concluded that the combination of navitoclax and irradiation exhibited a strong synergy regardless of the p53 mutation status.

### Navitoclax triggered apoptosis, which substantially increased after irradiation.

**Figure 4:**
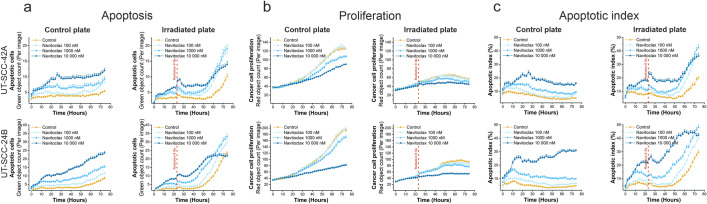
The navitoclax–irradiation combination triggers apoptosis and halts proliferation in HNSCC cells. The live cell apoptosis assay was performed for two cell lines (UT-SCC-42A and UT-SCC-24B). Cancer cells were labeled with CellTrace Far Red and the IncuCyte Caspase-3/7 Apoptosis Assay Reagent (green) was applied to detect apoptotic cells. Cells (1000 per well) were seeded to two plates and treated with three navitoclax concentrations (100, 1000 and 10,000 nM). The control group was treated with 0.1% DMSO. One of the plates was irradiated (8 Gy) after 24 h. Plates were imaged using the Incucyte S3 imaging system for a total of three days every other hour at 20 × objective (nine images per well). The number of apoptotic cells (green objects), the proliferation rate (red objects) and the percentage of apoptotic cells (green and red cells divided by red cells multiplied by 100) were calculated using the Incucyte analysis software. **(a)** Navitoclax induced apoptosis in both cell lines. The navitoclax–irradiation combination increased the number of apoptotic cells. **(b)** Navitoclax decreased proliferation in both cell lines, and after irradiation proliferation was effectively halted, particularly with 10,000-nM navitoclax. **(c)** Navitoclax elevated the apoptotic index in both cell lines. The apoptotic index increased rapidly while simultaneously halting proliferation 24 h after exposure to irradiation. In 48 h, the navitoclax–irradiation combination led to apoptosis in 40–50% of cells. Average ± SEM (n = 6).

Next, we performed a live cell apoptosis assay on two HNSCC cell lines. Navitoclax induced apoptosis, even as a single agent, dose dependently in both cell lines tested (Fig. 4a). Additionally, navitoclax reduced cancer cell proliferation in a dose-dependent manner (Fig. 4b). As expected, the navitoclax–irradiation combination led to a substantially elevated apoptosis rate and halted cancer cell proliferation in both cell lines (Fig. 4a,b). For the UT-SCC-42A cell line, the apoptotic cell number nearly doubled 48 h after the combination treatment compared with irradiation only (from 10 to 19 apoptotic cells per image) and quadrupled compared with untreated cells (from 5 to 19 apoptotic cells per image; Fig. 4a). For UT-SCC-24B, the apoptotic cell number increased by 43.5% (from 23 to 33 cells per image) following the combination treatment compared with irradiation only, and nearly quadrupled compared with untreated cells (from 9 to 33 cells per image; Fig. 4a). Combining 10,000-nM navitoclax and irradiation completely halted cell proliferation in both cell lines (Fig. 4b). For UT-SCC-42A, 10,000-nM navitoclax resulted in apoptosis in 30.1% of cells, which was six times higher compared with untreated cells (4.8%; Fig. 4c). The navitoclax–irradiation combination resulted in the highest apoptosis index (48.1%), which was 62.5% higher than with irradiation treatment (29.6%; Fig. 4c). For UT-SCC-24B, 10,000-nM navitoclax caused apoptosis in 15.8% of cells, which was three times higher compared with untreated cells (4.9%; Fig. 4c). The combination treatment resulted in an apoptosis index (42.4%) that was more than two times higher than with irradiation treatment (19.3%; Fig. 4c).

## Discussion

High-throughput compound screening (HTS) is a useful method for investigating effective cancer targeting compounds. We performed HTS using a library of 396 FDA-approved and investigational compounds and ionizing radiation as an initial screen to identify synergistic and antagonistic combinations for HPV-negative HNSCC cells cultured on a human tumor–derived Myogel matrix. Our screen revealed wide variation among the HNSCC cell lines tested in their responses to the majority of compounds and compound–irradiation combinations. Most compounds did not interact with irradiation (i.e., exhibited an additive effect), while some exhibited a strong synergy or antagonism. Following the initial screen, the most prominent compound–irradiation combinations were further investigated using five tenfold compound concentrations and four irradiation doses in the dose–response matrix analysis. We further investigated the most synergistic compound, navitoclax, using eight additional cell lines and a live cell apoptosis assay.

Multiple studies confirmed that radiosensitivity and radioresistance can depend on the schedule pattern. Although our study features only one type of schedule, we designed it to mimic the typical HNSCC treatment schedule, whereby radiotherapy begins simultaneously or shortly after drug administration. Furthermore, we chose to irradiate cells 24 h after cell seeding and drug administration to ensure complete cell attachment before irradiation.

Navitoclax is an orally active Blc-2 and Blc-xL inhibitor, which has exhibited in vitro activity against different tumor types as a single agent and together with chemotherapy^14^. However, only three in vitro studies exist for navitoclax in HNSCC^16, 17^, only one of which included navitoclax combined with irradiation in HNSCC cell lines^22^. In that study, Ow et al. found that this combination did not significantly improve the response and yielded only a modest benefit in two of eight cell lines^22^. Experiments were performed using a clonogenic survival assay with only one irradiation dose and two navitoclax concentrations. Several clinical trials have been conducted or are ongoing on navitoclax as a single agent or in combination with other anticancer compounds to treat leukemia and solid tumors. However, the navitoclax–radiotherapy combination remains clinically unexplored. Additionally, to our knowledge, no clinical trials exist for HNSCC. Thus, we report here for the first time a strong synergy between navitoclax combined with ionizing radiation in HNSCC cell lines (Fig. 3). Our dose–response matrix analysis confirmed a strong synergy in 10 HNSCC cell lines and a moderate synergy in another three cell lines. Thus, our findings encourage the clinical investigation of navitoclax in combination with irradiation for the treatment of HNSCC as well. Interestingly, other BH3 mimetics in our compound library tested appeared inefficacious (Supplementary Figure S1). The Bcl-2 selective inhibitor venetoclax appeared ineffective in all five cell lines as a single agent as previously reported^23^ and when combined with irradiation. The Bcl-2 and Mcl-1 inhibitor AT-101 exhibited a modest, although less convincing, synergy as the Bcl-2 and Bcl-xL inhibitor navitoclax. This indicates that the dual inhibition of Bcl-2 and Bcl-xL may play a crucial role in triggering apoptosis in HNSCC cells.

An inactivating mutation of p53 (the TP53 gene) is the most frequent genomic alteration in HNSCC, accounting for approximately 50% of cases^12^. A recent study reported that the combination of navitoclax with NOXA induction exhibits efficient anticancer effects in HNSCC cells regardless of the p53 status^17^. We selected six wild-type and six mutated p53 HNSCC cell lines to examine whether p53-mutated cell lines are less sensitive to Bcl-2/Bcl-xL inhibition and radiosynergy. Our screen demonstrated that navitoclax synergizes with radiotherapy regardless of the p53 mutation status (Fig. 3a). This indicates that navitoclax can bypass the survival advantage of the mutated p53 tumor suppressor gene following irradiation damage. However, as a single agent, navitoclax seemed more effective in p53 wild-type cell lines (Supplementary Figure S2).

The Bcl-2 family proteins regulate intrinsic (mitochondria-dependent) apoptosis in damaged cells by releasing a mitochondrial protein to cytosol, leading to the activation of caspase proteases^14^. Navitoclax induces intrinsic apoptosis in human tumor cells^15^. Our findings confirm that this phenomenon also occurs in HNSCC. Using a live cell apoptosis assay, we demonstrated that navitoclax triggers apoptosis in HNSCC cells (Fig. 4). In the presence of navitoclax, the apoptotic effect of irradiation increased markedly (Fig. 4). In addition, navitoclax reduced cancer cell proliferation as a single agent and arrested proliferation when combined with radiotherapy in both cell lines tested (Fig. 4).

In addition to navitoclax, our screen identified other promising radiosensitizers, including afatinib. Previous studies, which reported the radiosensitization of afatinib in HNSCC cell lines, support this finding^24, 25^. The first clinical trial to study afatinib in combination with radiation therapy in HNSCC is ongoing (NCT01783587). Furthermore, tretinoin exhibited a moderate synergy with irradiation in our screen, similar to a previous study in which tretinoin modulated radiosensitivity in HNSCC cell lines^26^. We also observed a radiosensitization of talazoparib, a PARP1/2 inhibitor, in two of our HNSCC cell lines, similar to findings among small cell lung cancer (SCLC) and some other solid tumors^27^. Several clinical trials combining talazoparib and radiotherapy are now recruiting lung, gynecological and breast cancer patients (NCT04170946, NCT03968406 and NCT04690855).

We also identified radioresistant properties among several drugs in HNSCC cell lines. Interestingly, two PLK1 inhibitors (BI 2536 and GSK-461364) showed strong antagonism when administered 24 h before irradiation. One study reported PLK1 inhibition causing radiosensitization or radioresistance depending on the treatment schedule in osteosarcoma and colorectal cancer cell lines using a clonogenic assay^28^. To date, no in vitro or clinical studies for HNSCC combined with BI 2536 and irradiation exist. Clinical trials for BI 2536 primarily focus on leukemia and solid tumors, such as breast, pancreatic, prostate and lung cancers. A phase II clinical trial for BI 2536 was completed for a panel of solid tumors, including HNSCC (NCT00526149). GSK-461364, an experimental compound, lacks in vitro studies for HNSCC. The only existing clinical trial for GSK-461364 was completed for non-Hodgkin's lymphoma (NCT00536835). Pemetrexed, a dihydrofolate reductase inhibitor, serves as treatment for pleural mesothelioma and non-small cell lung cancer (NSCLC). The pemetrexed–irradiation combination appears to carry schedule-dependent interactions in NSCLC and HNSCC cells^29^. In this study, 24-h incubation with pemetrexed in the HNSCC cell line (CAL-27) followed by irradiation (0–8 Gy) resulted in moderate antagonism^29^. Furthermore, pemetrexed was evaluated as a novel treatment for HNSCC patients. Although several clinical studies have been completed, the benefit of pemetrexed in HNSCC treatment remains unclear^30^. Surprisingly, based on our data, all cell lines sensitive to pemetrexed as a single agent exhibited a strong antagonism when combined with irradiation. This raises concerns regarding concomitant pemetrexed and radiotherapy in HNSCC.

Taken together, our HTS screen revealed both radiosensitive and radioresistant compounds for HNSCC cells. Pemetrexed and PLK1 inhibitors (BI 2536 and GSK-461364) exhibited a strong antagonism when combined with irradiation, whereas the combinations of irradiation with afatinib, tretinoin or talazoparib exhibited a promising synergy, in agreement with previous studies. The most promising finding—and, to our knowledge, the first such report—was that navitoclax combined with irradiation exhibited the strongest synergy, triggered apoptosis and halted the proliferation of HNSCC cells. Our findings, thus, encourage clinical trials using navitoclax combined with irradiation as treatment for HPV-negative HNSCC.

## Materials and methods

### Cell lines

We used 13 HPV-negative locally established HNSCC cell lines taken from primary and metastatic sites (Supplementary Table S2)^7, 31^. Among these, 12 UT-SCC cell lines were established in the Department of Otorhinolaryngology-Head and Neck Surgery at Turku University Hospital (Turku, Finland) and one cell line (OU-SCC-9B) was established at Oulu University Hospital (Oulu, Finland). The cells were cultured in a minimal essential medium (MEM; Gibco, Waltham, MA, USA) supplemented with l-glutamine (2 mmol/l), 10% fetal bovine serum, a nonessential amino acid solution, penicillin (100 U/ml), streptomycin (100 μg/ml) and 250-ng/ml fungizone (all from Sigma-Aldrich, St. Louis, MO, USA) at 37 °C in an atmosphere of 5% CO_2_. Cell lines were mycoplasma-free and tested using the PCR Mycoplasma Test Kit I/C (PromoKine, Heidelberg, Germany; cat no. PK-CA91-1048). Cell line authentication was not performed.

### Establishment of metastatic oral tongue squamous cell carcinoma cell line (OU-SCC-9B)

The Ethical Committee of the Northern Ostrobothnia Hospital District, Finland (statement number 31/2016) approved the study protocol, and all methods were performed in accordance with the relevant guidelines and regulations. To establish the metastatic oral tongue squamous cell carcinoma cell line (OU-SCC-9B; Supplementary Table S2), a piece of metastatic lymph node was obtained from a patient with oral cancer after they provided their informed consent. The fresh tissue was washed thoroughly in PBS containing 100-U/ml penicillin, 100-µg/ml streptomycin and 2.5-mg/ml amphotericin B (all from Sigma-Aldrich) and minced with a razor blade into small (1–2 mm) pieces. After washing with PBS, the tissue was digested in 1:1 Dulbecco’s Modified Eagle Medium (DMEM)/Ham´s Nutrient Mixture F 12 (Gibco) supplemented with 100-U/ml penicillin, 100-μg/ml streptomycin, 2.5-mg/ml amphotericin B and 1-mg/ml collagenase (all from Sigma-Aldrich) for about 1 h at 37 °C with stirring. After washing with PBS, single cells were obtained by filtering the samples through a 40-µm cell strainer (BD Biosciences, Franklin Lakes, NJ, USA). Cells were seeded on 12-well culture plates in MEM (Gibco) supplemented with 10% heat-inactivated FBS (Gibco), a 1% nonessential amino acid solution (Gibco), 2-mM glutamine, 100-U/ml penicillin, 100-µg/ml streptomycin and 250-ng/ml amphotericin B (all from Sigma-Aldrich). Fibroblasts were removed through brief exposure to a trypsin–EDTA solution (Sigma-Aldrich) as previously described^32^.

### High-throughput compound screen (HTS)

We used drug sensitivity and resistance testing (DSRT) adapted from a platform for leukemia cells^33^. We performed DSRT on HNSCC cell lines cultured in Myogel-coated wells on 384-well plates. Myogel was used to provide the TME for cancer cells, which improves the predictability of drug testing^7^. The use of human leiomyoma tissue was approved by the Ethics Committee of both Oulu and Tampere University Hospitals (statement number 2/2017), and all research was performed in accordance with relevant regulations. All prospective liquid handling was performed using an automated reagent dispenser (MultiFlo FX, BioTek, Winooski, Vermont, USA) and a HighRes Biosystems automation platform. Before screening, we performed irradiation dose optimization to determine the suitable dose (EC20 value) for each cell line. Next, we screened using the compound library applying a predefined irradiation dose (EC20). Compounds with the most synergistic and antagonistic effects were identified and further investigated using multiple doses of irradiation (0, 1, 2, 4 and 8 Gy).

### Compound library

We used a compound library of 396 FDA-approved drugs as well as investigational compounds and probes (Supplementary Figure S3). Most compounds were dissolved in dimethyl sulfoxide (DMSO) except for 19 compounds (e.g., some metabolic modifiers and platinum-based compounds), which were dissolved in water due to poor DMSO solubility or stability issues. The library covers kinase inhibitors and other signal transduction modulators. The majority of the compounds were investigational (46%) and FDA approved (46%; Supplementary Figure S3). A minority of the compounds (8%) were probes. A large proportion of the compounds consisted of kinase inhibitors (n = 208), differentiating/epigenetic modifiers (n = 73) or conventional chemotherapy drugs (n = 50). The library also contained small groups, such as hormone therapy drugs (n = 19), apoptotic modulators (n = 10), immunomodulatory compounds (n = 9) and rapalogs (n = 4). The library consisted of six compound plates (384-well plate, Corning, #3764) containing compounds at five concentrations. The compound plates were stored in pressurized inert nitrogen gas–filled storage pods (Roylan Developments Ltd., Surrey, UK) until used for screening. Each compound was tested over a 10 000-fold concentration range. DMSO at 0.1% served as the negative control and 100-μM Benzethonium chloride (BzCl) served as the positive control in the assay plates.

### Drug sensitivity workflow

To conduct drug sensitivity testing at the High Throughput Biomedicine Unit (HTB) at the Institute for Molecular Medicine Finland (FIMM), we used an automated system including ACell automation platform (HighRes Biosolutions, Beverly, MA, USA), a benchtop pipetting robot (Biomek FX, Beckman Coulter, Brea, USA), an ambient storage hotel (Cytomat 24, Thermo Scientific, Waltham, MA, USA) and an automated incubator (Cytomat 10C, Thermo Scientific). On the first day, Myogel was thawed on ice (4 °C) and diluted with a serum-free cell culture medium to 500 µg/ml. Diluted Myogel (500 µg/ml) was added to 384-well compound plates using a reagent dispenser (BioTek, MultiFlo FX). Plates were subsequently centrifuged for 20 s at 173×*g* at RT (Agilent VSpin, Santa Clara, CA, USA), and then placed in an automated incubator overnight. On the following day, cells were counted using the Scepter 2.0 Cell Counter (Merck Millipore, Burlington, MA, USA) and suspended to the desired density. The number of cells per well (500–750 cells/well) was optimized in our previous study^7^. Cells were seeded using a reagent dispenser (BioTek, MultiFlo FX, 20 µl/well) with a plate stacker, and the cells were left to adhere for 24 h in an automated incubator. After 24 h, plates were irradiated by gamma irradiator OB29/4 (STS, Braunschweig, Germany) using EC20 irradiation doses and placed back in an automated incubator. Control plates were also transferred to the gamma irradiator room to ensure similar handling for all plates. After 48 h, the CellTiter-Glo 2.0 (CTG) luminescent cell viability assay (Promega, Madison, WI, USA) was used to determine the amount of ATP in the assay wells. Plates were handled using the ACell platform allowing plates to cool (to RT) for 15 min, followed by dispensing 25-µl CTG to the assay wells using a Certus Flex dispenser (Fritz Gyger AG, Gwatt, Switzerland). The plates were centrifuged for 3 min at 1000 rpm at RT (Agilent VSpin, Santa Clara, CA, USA) and the luminescence signal was detected using the PHERAstar FS HT reader (BMG LABTECH GmbH, Ortenberg, Germany).

### Drug sensitivity testing data analysis

To quantitatively profile the compound effects, we calculated the drug sensitivity score (DSS) using the Breeze software (available at https://breeze.fimm.fi) ^34^. DSS was described in several previous studies^19, 33^, combining several parameters (IC50, the curve slope and the minimum and maximum responses) into a single metric. The compound effect was normalized against positive (100-μM Benzethonium Chloride, BzCl) and negative (0.1% DMSO) controls to calculate the dose–response curves for each compound in each cell line and condition (with and without irradiation) separately. Each cell line was screened using two parallel compound sets. One set served as a control (single agent) and the other set was irradiated with an optimized EC20 irradiation dose (compound–irradiation combination). Otherwise, plates were handled identically. To determine the synergistic and antagonistic effects of the compounds and irradiation, we calculated the delta DSS (ΔDSS) for each compound and cell line by subtracting the DSS for the compound–irradiation combination from the single agent DSS (Supplementary Figure S1). Depending on the positivity or negativity of the ΔDSS, the compounds were classified as synergistic or antagonistic, respectively. Based on the ΔDSS values, the most relevant synergistic and antagonistic compounds were selected for the validation experiment (Fig. 1).

### Dose–response matrix analysis and synergy scoring

For the synergy validation experiments, we used 384-well screening plates with 15 compounds in five concentrations in triplicate. Each cell line was seeded onto five plates, which were irradiated with different doses (0, 1, 2, 4 or 8 Gy). Otherwise, we used the same DSRT workflow as described above. To test whether the compound–irradiation combinations acted synergistically or antagonistically, we compared the observed responses to the expected combination responses. We then calculated the responses, and generated the dose–response matrices using the ZIP reference model with the SynergyFinder web application (version 2; synergyfinder.fimm.fi)^20, 21^. We further investigated the navitoclax–irradiation combination using eight additional UT-SCC cell lines. Based on the dose–response matrices and ZIP synergy scores, the compound–irradiation combinations were classified as noninteractive, antagonistic or synergistic. Combinations with a score > 10 were considered exhibiting a strong synergy and < − 10 a strong antagonism. Scores between 5 and 10 were considered moderately synergistic and between − 5 to − 10 as moderately antagonistic. Scores between − 5 and 5 were classified as noninteractive combinations.

### Live cell apoptosis assay

Two 96-well plates (PerkinElmer, #6005182, Waltham, MA, USA) were coated with 500-µg/ml Myogel (50-µl per well). Plates were then placed in an incubator overnight. On the following day, we prepared the cell suspension for the apoptosis assay using trypsin/EDTA to detach cells from the flask*.* The suspension of two cell lines (UT-SCC-42A and UT-SCC-24B) was labeled according to the manufacturer's instructions using CellTrace Far Red (Invitrogen, Carlsbad, CA, USA). One million cells were mixed with 1-ml PBS containing 1-µl CellTrace Far Red. Cells were placed in an incubator for 20 min at + 37 °C. Following incubation, a 5-ml culture medium was added, followed by 5-min incubation. Labeled cells were centrifuged for 5 min at 173×*g* at RT and suspended in a fresh culture medium. Cell suspension was diluted at a density of 100 000 cells/ml and divided into four groups: control with 0.1% DMSO and navitoclax concentrations (100, 1000 and 10,000 nM, respectively) in the presence of the IncuCyte Caspase-3/7 Apoptosis Assay Reagent (Sartorius; 1:1000). Cell suspension was pipetted onto the wells (1000 cells per well, 100 µl). Plates were placed in the Incucyte S3 live cell analysis system (Sartorius) and imaged every 2 h at objective 20× (9 images per well). Six replicates were used for each condition. After 24 h, plates were transferred to an irradiator room and one plate was irradiated with 8 Gy. After irradiation, the plates were placed back in Incucyte and imaged for 48 h. Cancer cell proliferation (red object count) and apoptotic cells (green object count) were quantified using the Incucyte analysis software. The apoptotic index (percentage of apoptotic cells) was determined by selecting apoptotic cells (green and red objects) divided by the total cell number (red object) multiplied by 100.

## Supplementary Information

Supplementary Figure S1.

Supplementary Figure S2.

Supplementary Figure S3.

Supplementary Tables.

## References

[1] Sung H (2021). Global cancer statistics 2020: GLOBOCAN estimates of incidence and mortality worldwide for 36 cancers in 185 countries. CA Cancer J. Clin..

[2] Siegel RL, Miller KD, Jemal A (2017). Cancer statistics, 2017. CA Cancer J. Clin..

[3] Wen Y, Grandis JR (2015). Emerging drugs for head and neck cancer. Expert Opin. Emerg. Drugs.

[4] Hutchinson L, Kirk R (2011). High drug attrition rates—Where are we going wrong?. Nat. Rev. Clin. Oncol..

[5] Salo T (2015). A novel human leiomyoma tissue derived matrix for cell culture studies. BMC Cancer.

[6] Salo T (2018). Organotypic three-dimensional assays based on human leiomyoma-derived matrices. Philos. Trans. R Soc. Lond. B Biol. Sci..

[7] Tuomainen K (2019). Human tumor-derived matrix improves the predictability of head and neck cancer drug testing. Cancers (Basel).

[8] Hanahan D, Weinberg RA (2011). Hallmarks of cancer: The next generation. Cell.

[9] Adams JM, Cory S (2007). The Bcl-2 apoptotic switch in cancer development and therapy. Oncogene.

[10] Kale J, Osterlund EJ, Andrews DW (2018). BCL-2 family proteins: Changing partners in the dance towards death. Cell Death Differ..

[11] Chang L (2014). PI3K/Akt/mTOR pathway inhibitors enhance radiosensitivity in radioresistant prostate cancer cells through inducing apoptosis, reducing autophagy, suppressing NHEJ and HR repair pathways. Cell Death Dis..

[12] Leemans CR, Braakhuis BJM, Brakenhoff RH (2011). The molecular biology of head and neck cancer. Nat. Rev. Cancer.

[13] Hemann MT, Lowe SW (2006). The p53-Bcl-2 connection. Cell Death Differ..

[14] Perini GF, Ribeiro GN, Pinto Neto JV, Campos LT, Hamerschlak N (2018). BCL-2 as therapeutic target for hematological malignancies. J. Hematol. Oncol..

[15] Tse C (2008). ABT-263: A potent and orally bioavailable Bcl-2 family inhibitor. Cancer Res..

[16] Ow TJ (2020). Apoptosis signaling molecules as treatment targets in head and neck squamous cell carcinoma. Laryngoscope.

[17] Britt EL (2019). Combination of fenretinide and ABT-263 induces apoptosis through NOXA for head and neck squamous cell carcinoma treatment. PLoS One.

[18] Lepikhova T (2018). Drug-sensitivity screening and genomic characterization of 45 HPV-negative head and neck carcinoma cell lines for novel biomarkers of drug efficacy. Mol. Cancer Ther..

[19] Yadav B (2014). Quantitative scoring of differential drug sensitivity for individually optimized anticancer therapies. Sci. Rep..

[20] He L (2018). Methods for high-throughput drug combination screening and synergy scoring. Methods Mol. Biol..

[21] Ianevski A, Giri AK, Aittokallio T (2020). SynergyFinder 2.0: Visual analytics of multi-drug combination synergies. Nucleic Acids Res..

[22] Ow TJ (2019). Optimal targeting of BCL-family proteins in head and neck squamous cell carcinoma requires inhibition of both BCL-xL and MCL-1. Oncotarget.

[23] Carter RJ (2019). Exploring the potential of BH3 mimetic therapy in squamous cell carcinoma of the head and neck. Cell Death Dis..

[24] Schütze C (2007). Combination of EGFR/HER2 tyrosine kinase inhibition by BIBW 2992 and BIBW 2669 with irradiation in FaDu human squamous cell carcinoma. Strahlenther. Onkol..

[25] Macha MA (2017). Afatinib radiosensitizes head and neck squamous cell carcinoma cells by targeting cancer stem cells. Oncotarget.

[26] Rossi L, Corvò R (2002). Retinoic acid modulates the radiosensitivity of head-and-neck squamous carcinoma cells grown in collagen gel. Int. J. Radiat. Oncol. Biol. Phys..

[27] Laird JH (2018). Talazoparib is a potent radiosensitizer in small cell lung cancer cell lines and xenografts. Clin. Cancer Res..

[28] Lund-Andersen C, Patzke S, Nähse-Kumpf V, Syljuåsen RG (2014). PLK1-inhibition can cause radiosensitization or radioresistance dependent on the treatment schedule. Radiother. Oncol..

[29] Wouters A (2010). In vitro study on the schedule-dependency of the interaction between pemetrexed, gemcitabine and irradiation in non-small cell lung cancer and head and neck cancer cells. BMC Cancer.

[30] Argiris A, Pennella E, Koustenis A, Hossain AM, Obasaju CK (2013). Pemetrexed in head and neck cancer: A systematic review. Oral Oncol..

[31] Stegeman H (2012). Activation of AKT by hypoxia: A potential target for hypoxic tumors of the head and neck. BMC Cancer.

[32] Carey TE (1983). Antibodies to human squamous cell carcinoma. Otolaryngol. Head Neck Surg..

[33] Pemovska T (2013). Individualized systems medicine strategy to tailor treatments for patients with chemorefractory acute myeloid leukemia. Cancer Discov..

[34] Potdar S (2020). Breeze: an integrated quality control and data analysis application for high-throughput drug screening. Bioinformatics.

