# Supplementary figures and images for: High-throughput compound screening identifies navitoclax combined with irradiation as a candidate therapy for HPV-negative head and neck squamous cell carcinoma
Source: Sci Rep. 2021 Jul 20;11:14755. doi: 10.1038/s41598-021-94259-5 (PMC8292418; doi:10.1038/s41598-021-94259-5)

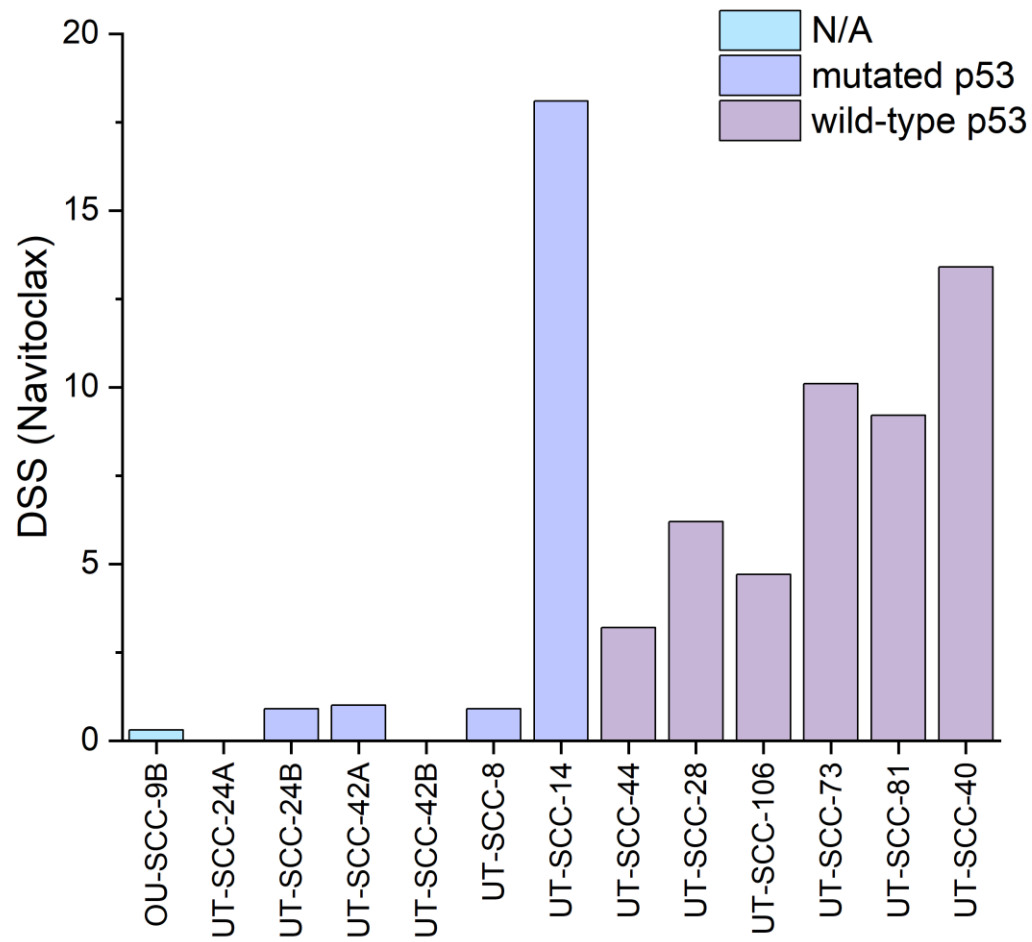

**Supplementary Figure S2.** The drug sensitivity scores (DSS) of navitoclax for 13 HNSCC cell lines.

Supplement: Supplementary file 2 — Supplementary Figure S2. [file 41598_2021_94259_MOESM2_ESM.pdf]
