# Supplementary material for: High-throughput compound screening identifies navitoclax combined with irradiation as a candidate therapy for HPV-negative head and neck squamous cell carcinoma
Source: Sci Rep. 2021 Jul 20;11:14755. doi: 10.1038/s41598-021-94259-5 (PMC8292418; doi:10.1038/s41598-021-94259-5)
Supplement: Supplementary file 3 — Supplementary Figure S3. [file 41598_2021_94259_MOESM3_ESM.pdf]

Supplementary Figure S3. The compound library of 396 FDA approved drugs, compounds and probes.

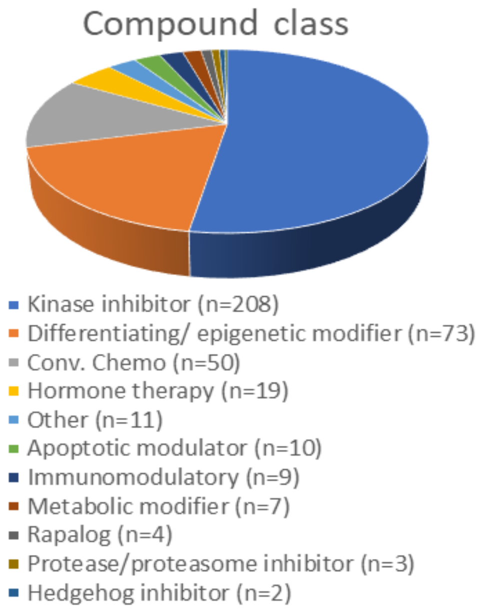

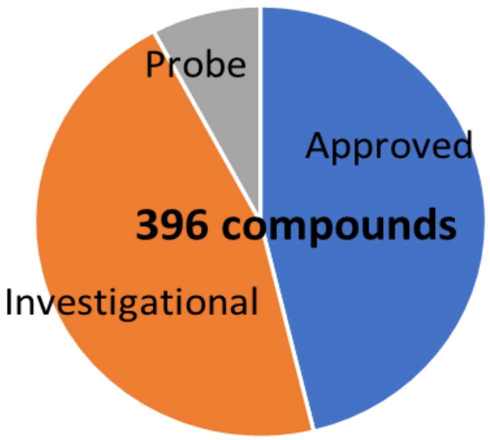

Drug	Mechanism/Targets	Class*	Phase/Approval status	Highest Conc (nM)
1-methyl-D-tryptophan	Indolamine 2,3-dioxygenase 1 and 2 inhibitor	X	Investigational (Ph 2)	5000
A-366	G9a/GLP inhibitor	E	Probe	25000
ABC294640	Sphingosine kinase 2 inhibitor	B	Investigational (Ph 2)	50000
Abemaciclib	CDK4/6 inhibitor	B	Approved	2500
Abexinostat	HDAC1-selective inhibitor	E	Investigational (Ph 2)	10000
Abiraterone	P450 17alpha-hydroxylase-17,20-lyase inhibitor	F	Approved	5000
Acalabrutinib	BTK inhibitor	B	Approved	1000
Acitretin	Retinoid receptor agonist	E	Approved (non-oncology)	10000
Afatinib	EGFR inhibitor	B	Approved	1000
Afuresertib	AKT1-selective inhibitor	B	Investigational (Ph 2)	1000
Alectinib	ALK (incl gatekeeper mut) inhib	B	Approved (Japan, US)	1000
Alisertib	Aurora A inhibitor	B	Investigational (Ph 3)	10000
Allopurinol	Xanthine oxidase inhibitor	A	Approved	10000
Alpelisib	PI3Kalpha selective inhibitor	B	Approved	2500
Alvocidib	CDK inhibitor	B	Investigational (Ph 2)	10000
Amcasertib	Cancer stem cell kinase inhibitor	B	Investigational (Ph 2)	10000
AMG-232	MDM2 inhibitor	G	Investigational (Ph 2)	10000
AMG319	PI3Kdelta inhibitor	B	Investigational (Ph 2)	1000
AMG-337	Met inhibitor	B	Investigational (Ph 2)	10000
Aminoglutethimide	Anti-steroid, aromatase inhibitor	F	Approved	10000
Amsacrine	DNA intercalation, Topo II inhibitor	A	Approved	10000
Amuvatinib	Broad spectrum TK inhib	B	Investigational (Ph 2)	10000
Anagrelide	PDE-3, PLA2 inhibitor	X	Approved	10000
Anastrozole	Aromatase inhibitor	F	Approved	10000
Apatinib	VEGFR inhibitor	B	Investigational (Ph 3)	10000
AR-42	HDAC inhibitor	E	Investigational (Ph 1)	10000
Arsenic(III) oxide	Thioredoxin reductase inhibitor	E	Approved	2500
ARV-825	BET-targeting PROTAC	E	Probe	300
ASP3026	ALK inhibitor	B	Investigational (Ph 1)	10000
AT 101	Bcl-2 family inhibitor	G	Investigational (Ph 2)	100000
AT13148	p70S6K, PKA, ROCK (AKT) inhibitor	B	Investigational (Ph 1)	10000
AT7519	CDK1, 2, 4, 6 and 9 inhibitor	B	Investigational (Ph 1)	10000
AT9283	Aurora A & B, Jak2, Flt, Abl inhibitor	B	Investigational (Ph 2)	1000
Atorvastatin	HMG-CoA reductase inhibitor	H	Approved	10000
Auranofin	Antirheumatic agent	A	Approved	2500
Axitinib	VEGFR, PDGFR, KIT inhibitor	B	Approved	10000
AZ 3146	Mps1 kinase (TTK) inhibitor	B	Probe	10000
Azacitidine	Nucleoside analog DNA methyl transferase inhibitor	E	Approved	10000
AZD-1080	GSK3 inhibitor	B	Investigational (Ph 1)	10000
AZD1152-HQPA	Aurora B inhibitor	B	Investigational (Ph 3)	1000
AZD1208	PIM1, 2, 3 kinase inhibitor	B	Investigational (Ph 1)	10000
AZD1480	JAK1/2, FGFR inhibitor	B	Investigational (Ph 1)	1000

AZD1775	Wee1 inhibitor	B	Investigational (Ph 2)	10000
AZD3759	EGFR inhibitor, BBB penetrable	B	Investigational (Ph 2)	1000
AZD4547	FGFR inhibitor	B	Investigational (Ph 2)	1000
AZD-5363	AKT inhibitor	B	Investigational (Ph 2)	10000
AZD-5438	CDK1,2,9 inhibitor	B	Investigational (Ph 1)	10000
AZD-6482	PI3Kbeta-selective inhibitor	B	Investigational (Ph 1)	2500
AZD7762	Chk1 inhibitor	B	Investigational (Ph 1)	1000
AZD8055	mTOR inhibitor	B	Investigational (Ph 1)	10000
AZD-8186	PI3Kbeta inhibitor	B	Investigational (Ph 1)	1000
Bafetinib	Abl, Lyn inhibitor	B	Investigational (Ph 2)	1000
Baricitinib	JAK inhibitor	B	Approved (EU)	2500
BAY 87-2243	HIF1alpha inhibitor	E	Investigational (Ph 1)	1000
BAY-1436032	IDH1 R132H/R132C inhibitor	E	Investigational (Ph 1)	10000
Belinostat	HDAC inhibitor	E	Approved (US)	10000
Bentamapimod	JNK inhibitor	B	Investigational (Ph 1)	10000
Bexarotene	Antineoplastic agent, retinoid	E	Approved	10000
BGB324	Axl inhibitor	B	Investigational (Ph 1)	10000
BI 2536	PLK1 inhibitor	B	Investigational (Ph 2)	1000
Bicalutamide	Nonsteroidal antiandrogen	F	Approved	10000
Bimatoprost	Prostaglandin analog	D	Approved	5500
Binimetinib	MEK1/2 inhibitor	B	Approved	1000
Birabresib	BET family inhibitor	E	Investigational (Ph 2)	10000
Birinapant	IAPs, SMAC mimetic	G	Investigational (Ph 2)	1000
Bleomycin	Glycopeptide antibiotic, causes DNA breaks	A	Approved	10000
BMS-754807	IGF1R inhibitor	B	Investigational (Ph 2)	10000
BMS-777607	Met, Axl, Ron and Tyro3 inhibitor	B	Investigational (Ph 2)	2500
BMS863233	Cdc7 inhibitor	B	Investigational (Ph 2)	10000
BMS-911543	JAK2 inhibitor	B	Investigational (Ph 1)	10000
Bortezomib	Proteasome inhibitor (26S subunit)	A	Approved	1000
Bosutinib	Abl, Src inhibitor	B	Approved	10000
Brigatinib	ALK inhibitor, including gatekeeper mutant ALK	B	Approved (US)	1000
Brivanib	VEGFR inhibitor	B	Investigational (Ph 3)	1000
Bryostatin 1	PKC activator	B	Investigational (Ph 1)	100
Buparlisib	PI3K inhibitor, pan-class I	B	Investigational (Ph 2)	10000
C646	p300/CREB-binding protein (CBP) inhibitor	E	Probe	25000
Cabazitaxel	Taxane microtubule stabilizer, antimitotic	A	Approved	1000
Cabozantinib	VEGFR2, Met, FLT3, Tie2, Kit and Ret inhibitor	B	Approved	1000
Canertinib	pan-HER inhibitor	B	Investigational (Ph 3)	10000
Capecitabine	5-FU prodrug	A	Approved	10000
Capmatinib	MET inhibitor	B	Approved	1000
Carboplatin	Platinum-based antineoplastic agent	A	Approved	100000
Carfilzomib	Proteasome inhibitor (20S subunit)	A	Approved	1000
CC-223	mTOR inhibitor	B	Investigational (Ph 2)	10000
Cediranib	KDR/Flt/VEGFR inhibitor	B	Investigational (Ph 3)	1000
Celecoxib	Selective COX-2 inhibitor	J	Approved	10000
CEP-32496	BRAF inhibitor	B	Investigational (Ph 2)	10000
CEP-37440	ALK inhibitor	B	Investigational (Ph 1)	5000
Ceritinib	ALK inhibitor	B	Approved	2500
Chloroquine	Antimalaria agent, chemo/radio sensitizer	A	Approved	100000
Cisplatin	Platinum-based antineoplastic agent	A	Approved	100000
Cladribine	Antimetabolite, Purine analog	A	Approved	1000
Clofarabine	Antimetabolite, Purine analog	A	Approved	10000
Clomifene	Selective estrogen receptor modulator	F	Approved	10000
Cobimetinib	MEK1/2 inhibitor	B	Approved (US)	1000
Copanlisib	PI3K alpha, delta selective inhibitor	B	Approved	1000
CPI-360	EZH2 inhibitor	E	Probe	10000
Crenolanib	PDGFRA and PDGFRB inhibitor	B	Investigational (Ph 2)	10000
Crizotinib	ALK, c-Met inhibitor	B	Approved	1000
CUDC-907	HDAC1/2/3/10, PI3Kalpha inhibitor	E	Investigational (Ph 2)	10000
Cytarabine	Antimetabolite, interferes with DNA synthesis	A	Approved	10000
Cytarabine/Idarubicin	Std. Induction therapy combination	A	Approved	5000/500
Dabrafenib	B-Raf(V600E) inhibitor	B	Approved	2500
Dacomitinib	pan-HER inhibitor	B	Approved	1000
Dactinomycin	RNA and DNA synthesis inhibitor	A	Approved	1000
Dactolisib	mTOR/(PI3K) inhibitor	B	Investigational (Ph 2)	1000
Danusertib	Aurora, Ret, TrkA, FGFR-1 inhibitor	B	Investigational (Ph 2)	10000
Dasatinib	Abl, Src, Kit, EphR... Inhibitor	B	Approved	1000
Daunorubicin	Topoisomerase II inhibitor	A	Approved	1000
dBET1	BET-targeting PROTAC	E	Probe	10000
Decernotinib	JAK3 inhibitor	B	Investigational (Ph 3)	10000
Decitabine	Nucleoside analog DNA methyl transferase inhibitor	E	Approved	10000
Deferoxamine	Iron chelator	X	Approved (non-oncology)	10000

Dexamethasone	Glucocorticoid, immunomodulatory agent	D	Approved	10000
Digoxin	Cardiac glycoside	X	Approved (non-oncology)	1000
Dinaciclib	CDK inhibitor	B	Investigational (Ph 3)	1000
Docetaxel	Mitotic inhibitor, taxane microtubule stabilizer	A	Approved	1000
Doramapimod	p38MAPK inhibitor	B	Investigational (Ph 1)	10000
Dovitinib	FGFR inhibitor	B	Investigational (Ph 3)	10000
Doxorubicin	Topoisomerase II inhibitor	A	Approved	1000
Duvelisib	PI3K inhibitor	B	Approved	500
Eltanexor	XPO1/CRM1 inhibitor	G	Investigational (Ph 2)	10000
Enasidenib	IDH2-R140Q inhibitor	E	Approved (US)	10000
Encorafenib	B-Raf(V600E) inhibitor	B	Approved	1000
ENMD-2076	pan-Aurora, VEGFR inhibitor	B	Investigational (Ph 2)	10000
Ensartinib	ALK inhibitor	B	Investigational (Ph 3)	1000
Entinostat	HDAC inhibitor	E	Investigational (Ph 2)	10000
Entospletinib	SYK inhibitor	B	Investigational (Ph 2)	5000
Entrectinib	TRK, ROS1, ALK inhibitor	B	Approved	1000
Enzalutamide	AR antagonist	F	Approved	10000
Enzastaurin	PKCbeta inhibitor	B	Investigational (Ph 3)	10000
Epirubicin	Topoisomerase II inhibitor	A	Approved	1000
EPZ015666	PRMT5 inhibitor	E	Probe	10000
EPZ031686	SMYD3 inhibitor	E	Probe	10000
EPZ-5687	EZH2 inhibitor	E	Probe	10000
Erdafitinib	FGFR inhibitor	B	Approved	1000
Eribulin	Mitotic inhibitor, microtubule depolymerizer.	A	Approved	1000
Erlotinib	EGFR inhibitor	B	Approved	10000
Etoposide	Topoisomerase II inhibitor	A	Approved	10000
Everolimus	binds FKBP12, causes inhibition of mTORC1	C	Approved	100
Exemestane	Aromatase inhibitor	F	Approved	10000
Fedratinib	JAK2-selective inhibitor	B	Approved	10000
Filgotinib	JAK1-selective inhibitor	B	Investigational (Ph 2)	10000
Finasteride	type II 5-alpha reductase inhibitor	F	Approved	10000
Fingolimod	S1PR antagonist	X	Approved	10000
Floxuridine	Antimetabolite, Analog of 5-fluorouracil	A	Approved	10000
Fludarabine	Antimetabolite, Purine analog	A	Approved	10000
Fluorouracil	Antimetabolite	A	Approved	10000
Flutamide	Nonsteroidal antiandrogen	F	Approved	10000
Foretinib	MET, VEGFR2 inhibitor	B	Investigational (Ph 2)	1000
Fostamatinib	Syk inhibitor	B	Approved	2500
Fulvestrant	Estrogen receptor antagonist	F	Approved	1000
Galunisertib	TGF-B/Smad inhibitor	B	Investigational (Ph 2)	1000
Gandotinib	JAK2 inhibitor	B	Investigational (Ph 2)	10000
GDC-0623	MEK1/2 inhibitor	B	Investigational (Ph 2)	2500
GDC-0853	BTK inhibitor	B	Investigational (Ph 2)	1000
Gedatolisib	PI3K/mTOR inhibitor	B	Investigational (Ph 2))	1000
Gefitinib	EGFR inhibitor	B	Approved	10000
Gemcitabine	Antimetabolite, Nucleoside analog	A	Approved	1000
Gilteritinib	FLT3/AXL inhibitor	B	Approved	1000
Givinostat	HDAC inhibitor	E	Investigational (Ph 2)	1000
Glesatinib	MET, AXL, TIE, VEGFR, RON inhibitor	B	Investigational (Ph 2)	2500
Golvatinib	MET, VEGFR2 inhibitor	B	Investigational (Ph 2)	2500
Goserelin	Gonadotropin releasing hormone superagonist	F	Approved	10000
GSK-1070916	AURb, AURc inhibitor	B	Investigational (Ph 1)	1000
GSK2256098	FAK inhibitor	B	Investigational (Ph 2)	1000
GSK2636771	PI3K beta selective inhibitor	B	Investigational (Ph 1)	10000
GSK2801	BAZ2B/A bromodomain inhibitor	E	Probe	10000
GSK2879552	LSD1 inhibitor	E	Investigational (Ph 1)	100000
GSK343	EZH2 inhibitor	E	Probe	1000
GSK-461364	PLK1 inhibitor	B	Investigational (Ph 1)	10000
GSK525762	BET family inhibitor	E	Investigational (Ph 1)	10000
GSK-690693	AKT, PKA, PKC inhibitor	B	Investigational (Ph 1)	10000
GSK-J4	JMJD3 (histone demethylase) inhibitor	E	Probe	100000
Hydroxyfasudil	ROCK, PKA, PKG, PRK inhibitor	B	Approved (Japan)	19000
Hydroxyurea	Antineoplastic agent	A	Approved	1000000
I-BET151	BET family inhibitor	E	Probe	10000
Ibrutinib	Btk inhibitor	B	Approved	1000
Icotinib	EGFR inhibitor	B	Investigational (Ph 2)	10000
Idarubicin	Topoisomerase II inhibitor	A	Approved	1000
Idasanutlin	p53-MDM2 inhibitor	G	Investigational (Ph 3)	10000
Idelalisib	PI3K inhibitor, p110δ-selective	B	Approved	10000
Imatinib	Abl, Kit, PDGFRB inhibitor	B	Approved	10000
Imiquimod	Immunomodulatory agent, TLR7 agonist	D	Approved	2500
IOX-1	2-Oxoglutarate Oxygenase Inhibitor	E	Probe	100000

IOX-2	PHD2 inhibitor	E	Probe	50000
Ipatasertib	AKT inhibitor	B	Investigational (Ph 2)	10000
Itraconazole	antifungal, hedgehog signaling inhibitor	X	Approved (non-oncology)	5000
Ivosidenib	IDH1 R132H/R132C inhibitor	E	Approved	10000
Ixabepilone	Mitotic inhibitor. Epothilone microtubule stabilizer.	A	Approved	1000
Ixazomib	Proteasome inhibitor (20S subunit)	A	Approved	1000
JQ1	BET family inhibitor	E	Probe	10000
KD025	ROCK2 inhibitor	B	Investigational (Ph 2)	5000
Lapatinib	HER2, EGFR inhibitor	B	Approved	1000
Larotrectinib	TRK inhibitor	B	Approved	1000
Lasofoxifene	Selective estrogen receptor modulator	F	Approved	1000
Lenalidomide	Immunomodulatory	D	Approved	100000
Lenvatinib	VEGFR inhibitor	B	Approved (US)	2500
Letrozole	Aromatase inhibitor	F	Approved	10000
Linifanib	VEGFR, PDGFR, CSF-1R, FLT3 inhibitor	B	Investigational (Ph 3)	1000
Linsitinib	IGF1R, IR inhibitor	B	Investigational (Ph 2)	10000
Lomeguatrib	O6-methylguanine-DNA methyltransferase inhibitor	E	Investigational (Ph 2)	10000
Lonafarnib	Farnesyltransferase inhibitor	E	Approved	100000
Losmapimod	p38MAPK inhibitor	B	Investigational (Ph 3)	10000
Lovastatin	HMG-CoA reductase inhibitor	H	Approved (non-oncology)	10000
Lucitanib	FGFR1, VEGFR inhibitor	B	Investigational (Ph 2)	10000
LY-2874455	FGFR inhibitor	B	Investigational (Ph 1)	1000
LY3023414	PI3K/mTOR/DNA-PK inhibitor	B	Investigational (Ph 2)	2500
Masitinib	KIT inhibitor	B	Investigational (Ph 3)	10000
Megestrol acetate	Progestogen	F	Approved	10000
Mepacrine	Unclear. PLA2 inhibitor. NF-kB inhibitor, p53 activator	X	Approved	50000
Mercaptopurine	Antimetabolite	A	Approved	10000
Merestinib	Met inhibitor	B	Investigational (Ph 2)	1000
Metformin	AMPK activator	H	Approved (non-oncology)	100000
Methotrexate	Antimetabolite, Anti-folate agent	H	Approved	5000
Methylprednisolone	Glucocorticoid, immunomodulatory agent	D	Approved	10000
Midostaurin	Broad TK (FLT3, KIT, RET, JAK, EGFR...) inhibitor	B	Approved	10000
Milciclib	CDK2 inhibitor	B	Investigational (Ph 2)	10000
Miltefosine	Antimicrobial, inhibits PI3K/AKT	X	Approved	100000
Mitomycin C	Antineoplastic anatibiotic, DNA crosslinker	A	Approved	10000
Mitotane	Antineoplastic agent	A	Approved	10000
Mitoxantrone	Topoisomerase II inhibitor	A	Approved	1000
Mivebresib	BET family inhibitor	E	Investigational (Ph 1)	10000
MK-2206	AKT inhibitor	B	Investigational (Ph 2)	1000
MK-8776	CHEK1 inhibitor	B	Investigational (Ph 1)	2500
ML390	DHODH inhibitor	E	Probe	50000
MLN-0128	mTOR inhibitor	B	Investigational (Ph 1)	1000
MLN1117	PI3Kalpha selective inhibitor	B	Investigational (Ph 2)	10000
Mocetinostat	HDAC inhibitor (HDAC1 & 2-selective)	E	Investigational (Ph 2)	10000
Momelotinib	JAK1 & 2 inhibitor	B	Investigational (Ph 2)	10000
Motesanib	VEGFR, PDGFR, Ret, Kit inhibitor	B	Investigational (Ph 2)	10000
Mubritinib	HER2 inhibitor	B	Investigational (Ph 1)	1000
Navitoclax	Bcl-2/Bcl-xL inhibitor	G	Investigational (Ph 2)	10000
Nelarabine	Nucleoside analog, DNA, RNA synth inhibitor	A	Approved	10000
Neratinib	HER2, EGFR inhibitor	B	Approved (US)	1000
Nilotinib	Abl inhibitor	B	Approved	10000
Nilutamide	Nonsteroidal antiandrogen	F	Approved	10000
Nintedanib	VEGFR, PDGFR, FGFR inhibitor	B	Approved	10000
Niraparib	PARP inhibitor	E	Approved (US)	10000
NVP-AEW541	IGF1R inhibitor	B	Investigational (Ph 1)	10000
NVP-BGJ398	FGFR inhibitor	B	Investigational (Ph 1)	1000
NVP-BGT226	PI3K/mTOR inhibitor	B	Investigational (Ph 2)	1000
NVP-LCL161	IAPs, SMAC mimetic	G	Investigational (Ph 2)	25000
NVP-RAF265	C-Raf inhibitor, unclear MoA	B	Investigational (Ph 2)	1000
Olaparib	PARP inhibitor	E	Approved	10000
Olmutinib	EGFR(L858R/T790M) inhibitor	B	Investigational (Ph 2)	1000
Omacetaxine	Protein synthesis inhib (80 S ribosome)	A	Approved	10000
Omipalisib	PI3K/mTOR inhibitor	B	Investigational (Ph 1)	1000
Osimertinib	EGFR(L858R/T790M) inhibitor	B	Approved	2500
OSU-03012	PDPK1 inhibitor	B	Investigational (Ph 1)	25000
OTS167	MELK inhibitor	B	Investigational (Ph 2)	1000
Oxaliplatin	Platinum-based antineoplastic agent	A	Approved	100000
Paclitaxel	Mitotic inhibitor, taxane microtubule stabilizer	A	Approved	1000
Pacritinib	FLT3/JAK2	B	Investigational (Ph 3)	10000
Palbociclib	CDK4/6 inhibitor	B	Approved	10000
Palomid-529	AKT, MTOR, PI3K inhibitor	B	Investigational (Ph 1)	10000
Panobinostat	HDAC inhibitor	E	Approved	1000

Pazopanib	VEGFR inhibitor	B	Approved	10000
PCI-34051	HDAC8 inhibitor	E	Probe	10000
PD0325901	MEK1/2 inhibitor	B	Investigational (Ph 2)	1000
Peficitinb	JAK3-selective inhibitor	B	Investigational (Ph 3)	2500
Pemetrexed	Dihydrofolate reductase inhibitor	H	Approved	10000
Pentostatin	Antimetabolite, Purine analog	A	Approved	10000
Perifosine	AKT/PI3K inhibitor	B	Investigational (Ph 3)	2500
Pexidartinib	KIT, CSF1R, FLT3 inhibitor	B	Approved	10000
PF-00477736	Chk1 inhibitor	B	Investigational (Ph 1)	10000
PF-00562271	FAK inhibitor	B	Investigational (Ph 1)	10000
PF-03758309	PAK inhibitor	B	Investigational (Ph 1)	10000
PF-06463922	ALK, ROS1 inhibitor	B	Investigational (Ph 2)	1000
PF06650833	IRAK4 inhibitor	B	Investigational (Ph 2)	10000
PFI-1	BET family inhibitor	E	Probe	30000
PH-797804	p38MAPK inhibitor	B	Investigational (Ph 2)	1000
Pictilisib	PI3K inhibitor, pan-class I	B	Investigational (Ph 2)	10000
Pilocarpine	Non-selective muscarinic receptor agonist	X	Approved	40000
Pinometostat	DOT1L inhibitor	E	Investigational (Ph 1)	1000
Pirfenidone	Antifibrotic and anti-inflammatory	D	Approved	10000
Pixantrone	topoisomerase II inhibitor	A	Investigational (Ph 3)	10000
Plerixafor	CXCR4 antagonist	X	Approved	10000
Plicamycin	RNA synthesis inhibitor	A	Approved	10000
Pomalidomide	Immunomodulatory agent, anti-angiogenic	D	Approved	10000
Ponatinib	Broad TK inhibitor	B	Approved	1000
Poziotinib	pan-HER inhibitor	B	Investigational (Ph 2)	1000
Pracinostat	HDAC inhibitor	E	Investigational (Ph 2)	10000
Pravastatin	HMG-CoA reductase inhibitor	H	Approved (non-oncology)	10000
Prednisolone	Glucocorticoid, immunomodulatory agent	D	Approved	10000
Prexasertib	Chk1 inhibitor	B	Investigational (Ph 2)	10000
PTC-209	BMI-1 inhibitor	E	Probe	10000
Quisinostat	HDAC inhibitor	E	Investigational (Ph 2)	1000
Quizartinib	FLT3 inhibitor	B	Investigational (Ph 3)	1000
Rabusertib	Chk1 inhibitor	B	Investigational (Ph 2)	1000
Radotinib	ABL, PDGFR inhibitor	B	Investigational (Ph 3)	10000
Ralimetinib	p38MAPK inhibitor	B	Investigational (Ph 2)	10000
Raloxifene	Selective estrogen receptor modulator	F	Approved	10000
Raltitrexed	DHFR/GARFT/thymidylate synthase inhibitor	A	Approved	1000
Regorafenib	B-Raf, c-Kit, VEGFR2 inhibitor	B	Approved	10000
Resminostat	HDAC1, 3, 6 inhibitor	E	Investigational (Ph 2)	10000
RGFP966	HDAC3 inhibitor	E	Probe	10000
Ribociclib	CDK4/6 inhibitor	B	Approved (US)	10000
Ripasudil	ROCK inhibitor	B	Approved (Japan)	10000
Rociletinib	EGFR(L858R/T790M) inhibitor	B	Investigational (Ph 3)	10000
Rocilinostat	HDAC-6 selective inhibitor	E	Investigational (Ph 1)	10000
Romidepsin	HDAC inhibitor	E	Approved	1000
Roxadustat	HIF prolyl hydroxylase inhibitor	E	Investigational (Ph 2)	10000
Ruboxistaurin	PKCbeta inhibitor	B	Investigational (Ph 3)	10000
Rucaparib	PARP inhibitor	E	Approved (US)	10000
Ruxolitinib	JAK1&2 inhibitor	B	Approved	10000
Salinomycin	Ionophore	X	Veterinary approval	50000
Sapitinib	Pan-HER inhibitor	B	Investigational (Ph 2)	1000
Saracatinib	Src, Abl inhibitor	B	Investigational (Ph 3)	10000
Seliciclib	CDK2/7/9 inhibitor	B	Investigational (Ph 2)	10000
Selinexor	XPO1/CRM1 inhibitor	G	Approved	10000
Selonsertib	ASK1 inhibitor	B	Investigational (Ph 2)	1000
Selumetinib	MEK1/2 inhibitor	B	Approved	10000
Sepantronium bromide	Survivin inhibitor	G	Investigational (Ph 2)	10000
SGC0946	DOT1L inhibitor	E	Probe	10000
SGC-CBP30	CREBBP/EP300 bromodomain inhibitor	E	Probe	25000
SGI-1776	PIM kinase inhibitor	B	Investigational (Ph 1)	10000
Silmitasertib	CSNK2A1 inhibitor	B	Investigational (Ph 2)	10000
Simvastatin	HMG-CoA reductase inhibitor	H	Approved (non-oncology)	10000
Sirolimus	binds FKBP12, causes inhibition of mTORC1	C	Approved	100
SN-38	Topoisomerase I inhibitor	A	(Approved)	10000
SNS-032	CDK inhibitor	B	Investigational (Ph 2)	10000
Sonidegib	Smoothened (Hh) inhibitor	X	Approved	10000
Sonolisib	PI3K inhibitor, pan-class I. Irreversible	B	Investigational (Ph 2)	10000
Sorafenib	B-Raf, FGFR-1, VEGFR-2 & -3, PDGFR-beta inhib	B	Approved	1000
Sotrastaurin	PKC inhibitor	B	Investigational (Ph 2)	10000
Spebrutinib	BTK inhibitor	B	Investigational (Ph 2)	1000
StemRegenin 1	AHR antagonist, stem cell regenerating	E	Probe	10000
Sunitinib	Broad TK inhibitor	B	Approved	1000

Tacedinaline	HDAC inhibitor	E	Investigational (Ph 3)	1000
Tacrolimus	Binds FKBP12, causes inhibition of calcineurin	C	Approved	10000
TAK-285	HER2 inhibitor	B	Investigational (Ph 1)	2500
TAK-901	Aurora, Src family, JAK3, RTK inhibitor	B	Investigational (Ph 1)	1000
Talazoparib	PARP1/2 inhibitor	E	Approved	1000
Talmapimod	p38MAPK alpha selective inhibitor	B	Investigational (Ph 2)	10000
Tamatinib	Syk inhibitor	B	Approved	10000
Tamoxifen	Estrogen receptor antagonist	F	Approved	10000
Tandutinib	FLT3, PDGFR, KIT inhibitor	B	Investigational (Ph 2)	1000
Tanzisertib	JNK inhibitor	B	Investigational (Ph 2)	10000
Taselisib	PI3K alpha, delta, (gamma) selective inhibitor	B	Investigational (Ph 3)	1000
Tazemetostat	EZH2 inhibitor	E	Approved	10000
Telatinib	VEGFR, KIT, PDGFR inhibitor	B	Investigational (Ph 2)	10000
Temozolomide	Alkylating agent	A	Approved	100000
Temsirolimus	binds FKBP12, causes inhibition of mTORC1	C	Approved	100
Teniposide	Topoisomerase II inhibitor	A	Approved	10000
Tepotinib	c-Met inhibitor	B	Approved	1000
Tesevatinib	EGFR, ERBB2, VEGFR, EPHB4	B	Investigational (Ph 2)	1000
TG100-115	PI3K gamma/delta inhibitor	B	Investigational (Ph 2)	10000
TGR-1202	PI3Kdelta inhibitor	B	Investigational (Ph 3)	2500
Thalidomide	Immunosuppressant	D	Approved	10000
Thioguanine	Antimetabolite, Purine analog	A	Approved	10000
Tideglusib	GSK3 inhibitor	B	Investigational (Ph 2)	3000
Tipifarnib	Farnesyltransferase inhibitor	E	Investigational (Ph 3)	10000
Tivantinib	MET inhibitor	B	Investigational (Ph 2)	1000
Tivozanib	VEGFR1, 2, 3, c-Kit, PDGFRB inhibitor	B	Investigational (Ph 3)	10000
Tofacitinib	JAK3, JAK2(V617F) inhibitor	B	Approved	5000
Topotecan	Topoisomerase I inhibitor. Camptothecin analog	A	Approved	10000
Toremifene	selective estrogen receptor modulator	F	Approved	10000
Tozasertib	pan-Aurora inhibitor	B	Investigational (Ph 2)	10000
Trametinib	MEK1/2 inhibitor	B	Approved	250
Tretinoin	Retinoic acid receptor agonist	E	Approved	10000
Triciribine	AKT inhibitor	B	Investigational (Ph 2)	100000
Trifluridine	Antimetabolite, Nucleoside analog	A	Approved	10000
Tubacin	HDAC6 inhibitor	E	Probe	10000
Tubastatin A	HDAC6 inhibitor	E	Probe	10000
Tucatinib	HER2 inhibitor	B	Approved	2500
Tucidinostat	HDAC1/2/3/10 inhibitor	E	Investigational (Ph 2)	10000
UCN-01	PKCbeta, PDK1, Chk, Cdk2 inhibitor	B	Investigational (Ph 2)	10000
Ulixertinib	ERK inhibitor	B	Investigational (Ph 2)	10000
UM729	Enhancer of aryl hydrocarbon receptor antagonists	E	Probe	10000
UNC0638	G9a/GLP inhibitor	E	Probe	10000
UNC0642	G9a/GLP inhibitor	E	Probe	10000
UNC1215	L3MBTL3 inhibitor	E	Probe	10000
Upadacitinib	JAK1-selective inhibitor	B	Approved	10000
Uprosertib	AKT inhibitor	B	Investigational (Ph 2)	10000
Valproic acid	HDAC inhibitor	E	Approved	1000000
Valrubicin	Topoisomerase II inhibitor	A	Approved	5000
Vandetanib	VEGFR,EGFR, RET inhibitor	B	Approved	1000
Varlitinib	EGFR HER2 inhibitor	B	Investigational (Ph 2)	10000
Vatalanib	VEGFR-1 & -2 inhibitor	B	Investigational (Ph 3)	10000
Veliparib	PARP inhibitor	E	Investigational (Ph 3)	10000
Vemurafenib	B-Raf(V600E) inhibitor	B	Approved	10000
Venetoclax	Bcl-2-selective inhibitor	G	Approved (US)	1000
Vidofludimus	DHODH inhibitor	E	Investigational (Ph 2)	10000
Vinblastine	Mitotic inhib.Vinca alkaloid microtubule depolymerizer	A	Approved	1000
Vincristine	Mitotic inhib.Vinca alkaloid microtubule depolymerizer	A	Approved	1000
Vinflunine	Mitotic inhib.Vinca alkaloid microtubule depolymerizer	A	Approved	1000
Vinorelbine	Mitotic inhib.Vinca alkaloid microtubule depolymerizer	A	Approved	10000
Vismodegib	Smoothened (Hh) inhibitor	X	Approved	10000
Vistusertib	mTOR inhibitor, ATP-competitive	B	Investigational (Ph 2)	10000
Volasertib	PLK1 inhibitor	B	Investigational (Ph 3)	1000
Vorinostat	HDAC inhibitor	E	Approved	10000
VX-745	p38MAPK inhibitor	B	Investigational (Ph 2)	10000
XAV-939	Tankyrase-1 and -2	E	Probe	10000
ZSTK474	PI3K gamma selective inhibitor	B	Investigational (Ph 1)	10000

*A= Conv. Chemo, B=Kinase inhibitor, C=Rapalog, D= Immunomodulatory, E= Differentiating/ epigenetic modifier, F=Hormone therapy, G= Apoptotic modulator, H= Metabolic modifier, J= NSAID, L=Protease/proteasome inhibitor, X= Other
