# Supplementary material for: High-throughput compound screening identifies navitoclax combined with irradiation as a candidate therapy for HPV-negative head and neck squamous cell carcinoma
Source: Sci Rep. 2021 Jul 20;11:14755. doi: 10.1038/s41598-021-94259-5 (PMC8292418; doi:10.1038/s41598-021-94259-5)
Supplement: Supplementary file 4 — Supplementary Tables. [file 41598_2021_94259_MOESM4_ESM.pdf]

Supplementary Table 1. The synergy scores and most synergistic are scores for each 15 compounds for five cell lines.

		UT-SCC-24A		UT-SCC-24B		UT-SCC-42A		UT-SCC-42B		OU-SCC-9B	
**Drug**	**Molecular target**	**Synergy score**	**The most synergistic are score**	**Synergy score**	**The most synergistic are score**	**Synergy score**	**The most synergistic are score**	**Synergy score**	**The most synergistic are score**	**Synergy score**	**The most synergistic are score**
**Birinapant**	IAPs, SMAC mimetic	1,64	3,50	4,57	11,36	0,18	0,66	-0,02	0,00	0,57	1,23
**Talazoparib**	PARP1/2 inhibitor	1,18	3,10	6,73	11,55	3,15	7,33	0,42	0,92	-0,02	0,00
**Tretinoin**	Retinoic acid receptor agonist	-0,02	0,00	1,81	5,11	4,06	9,17	-0,02	0,00	0,73	1,59
**Afatinib**	EGFR inhibitor	1,52	3,55	0,21	0,50	4,51	9,90	0,66	2,35	-2,25	0,82
**Pemetrexed**	Dihydrofolate reductase inhibitor	-4,33	0,00	-20,06	0,04	-28,33	0,77	-0,02	0,00	-0,02	0,00
**BI 2536**	PLK1 inhibitor	-4,05	4,83	-4,36	0,12	-18,48	2,09	-14,39	4,18	0,34	1,02
**Navitoclax**	Bcl-2/Bcl-xL inhibitor	3,63	9,48	7,73	18,60	11,19	23,24	11,85	26,17	3,31	6,93
**Lonafarnib**	Farnesyltransferas e inhibitor	1,13	2,58	-0,19	1,03	0,18	0,81	-1,83	0,45	1,37	3,10
**Triciribine**	AKT inhibitor	1,86	2,54	1,92	4,13	2,57	3,31	1,51	2,88	-0,01	1,97
**Omipalisib**	PI3K/mTOR inhibitor	1,22	1,93	1,84	3,20	2,80	4,93	0,92	2,25	0,70	2,29
**CUDC-907**	HDAC1/2/3/10, PI3Kalpha inhibitor	0,34	0,62	-0,03	0,11	-0,14	0,17	-0,19	0,17	0,72	1,43
**GSK-461364**	PLK1 inhibitor	-6,05	1,10	-5,67	2,29	-16,56	-1,12	-14,97	5,34	-0,17	0,00
**Tipifarnib**	Farnesyltransferas e inhibitor	-0,44	0,02	-1,64	0,14	0,34	1,28	-1,59	0,32	1,46	3,81
**Acitretin**	Retinoid receptor agonist	0,28	1,25	0,97	2,73	0,82	1,75	-0,02	0,00	0,23	0,52
**BAY 87-2243**	HIF1alpha inhibitor	2,62	5,42	2,51	6,15	-0,53	0,07	-1,28	0,07	0,86	2,91

Supplementary Table 2. Clinical and pathological characteristics of the HNSCC cell lines. TNM is based on the pathology report.

Cell line	Sex[a]	Age[b]	TNM	Specimen site	Type[c]	Grade	P53 status
**UT-SCC-8**	M	42	T2N0M0	larynx	pri	G1	non-synonomous SNV
**UT-SCC-14**	M	25	T3N1M0	tongue	pri(per)	G2	splicing mutation
**UT-SCC-24A**	M	41	T2N0M0	tongue	pri	G2	splicing mutation
**UT-SCC-24B**	M	41	T2N1M0	neck	met(per)	G2	splicing mutation
**UT-SCC-28**	F	48	T2N0M0	floor of mouth	pri(per)	G1	wild type
**UT-SCC-42A**	M	43	T4N3M0	larynx	pri	G3	stop-gain mutation
**UT-SCC-42B**	M	43	T4N3M0	neck	met	G3	stop-gain mutation
**UT-SCC-40**	M	65	T3N0M0	tongue	pri	G1	wild type
**UT-SCC-44**	F	71	T4N2BM0	gingiva of mandibula	pri(per)	G3	wild type
**UT-SCC-73**	F	86	T1N0M0	tongue	pri	G2	wild type
**UT-SCC-81**	M	48	T2N0M0	tongue	pri	G1	wild type
**UT-SCC-106A**	M	37	T1AN0M0	larynx	pri	G1	wild type
**OU-SCC-9B**	M	55	T4N3M1a	neck*	met	G2	N/A

[a]M=male, F=female, [b] Age in years, [c] Pri=primary tumor, met=metastasis, per= persistent disease
***primary tumor location: tongue**
