# Supplementary material for: High-throughput compound screening identifies navitoclax combined with irradiation as a candidate therapy for HPV-negative head and neck squamous cell carcinoma
Source: Sci Rep. 2021 Jul 20;11:14755. doi: 10.1038/s41598-021-94259-5 (PMC8292418; doi:10.1038/s41598-021-94259-5)
Supplement: Supplementary file 1 — Supplementary Figure S1. [file 41598_2021_94259_MOESM1_ESM.pdf]

Supplementary Figure S1. The heatmaps of the DSSs and the ΔDSSs for five cell lines and for each compound. The heatmaps were created with the help of Breeze DSRT pipeline (https://breeze.fimm.fi)[34]. The pipeline and scripts used for the drug sensitivity profiles were developed using the R programming language.

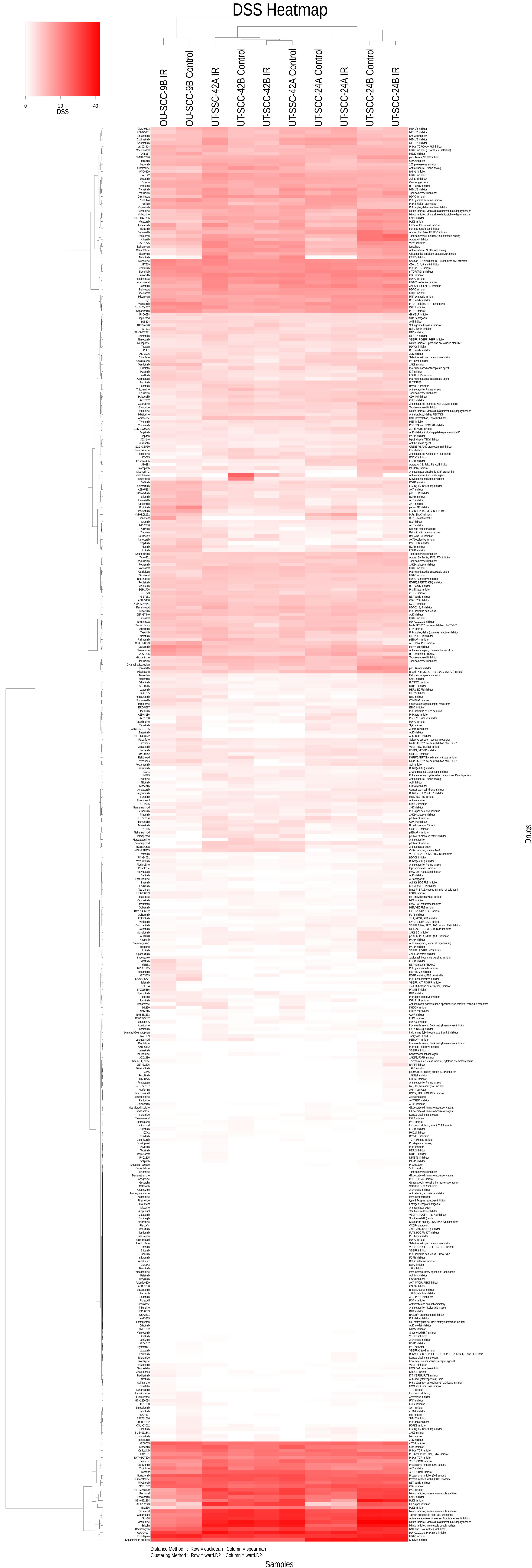

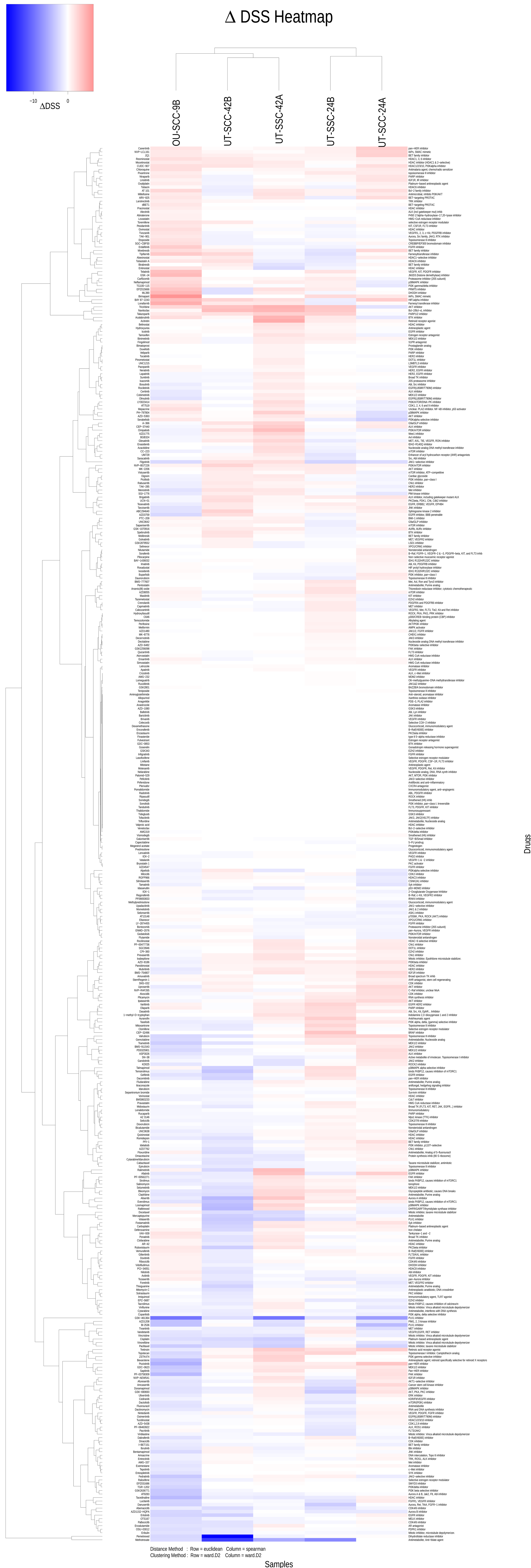
Δ DSS Heatmap
−10
0
ΔDSS
OU-SCC-9B
UT-SCC-42B
UT-SSC-42A
UT-SSC-24B
UT-SCC-24A
Drugs
Distance Method : Row = euclidean Column = spearman
Clustering Method : Row = ward.D2 Column = ward.D2
Samples
